# miR-495-3p depresses cell proliferation and migration by downregulating HMGB1 in colorectal cancer

**DOI:** 10.1186/s12957-022-02500-w

**Published:** 2022-03-30

**Authors:** Jie Ling Zhang, Hui Fen Zheng, Kai Li, Yi Ping Zhu

**Affiliations:** 1 Department of Oncology, The First Affiliated Hospital of Wannan Medical College, Wuhu, 241002 China; 2 Department of Clinical Medicine, Wannan Medical College, Wuhu, 241002 China; 3 Anhui Province Key Laboratory of Biological Macro-molecules Research, Wannan Medical College, Wuhu, 241002 China

**Keywords:** miR-495-3p, HMGB1, Colorectal cancer, Proliferation, Migration

## Abstract

**Background:**

MicroRNAs play an important role in the genesis and progression of tumours, including colorectal cancer (CRC), which has a high morbidity and mortality rate. In this research, the role of miR-495-3p and HMGB1 in CRC was investigated.

**Methods:**

We performed qRT-PCR to detect the expression of miR-495-3p in colorectal cancer tissues and cell lines. Functional experiments, such as CCK-8, EdU, Transwell and apoptosis assays, were conducted to explore the effects of miR-495-3p on the proliferation, migration and apoptosis of CRC cells in vitro. Then, database prediction, dual-luciferase reporter gene assays and functional experiments verified the role of the miR-495-3p target gene HMGB1 in CRC. Finally, rescue experiments were performed to investigate whether overexpression of HMGB1 could reverse the inhibitory effect of miR-495-3p on CRC cell proliferation in vivo and in vitro.

**Results:**

miR-495-3p was downregulated in colorectal cancer tissues and cell lines, inhibited the proliferation and migration of colorectal cancer cells and promoted cell apoptosis. Database prediction and dual-luciferase reporter gene assays showed that HMGB1 was the downstream target gene of miR-495-3p. We finally demonstrated that miR-495-3p inhibited CRC cell proliferation by targeting HMGB1 in vitro and in vivo.

**Conclusion:**

Our research shows that miR-495-3p inhibits the progression of colorectal cancer by downregulating the expression of HMGB1, which indicates that miR-495-3p may become a potential therapeutic target for colorectal cancer.

## Introduction

Colorectal cancer (CRC) is the third most common malignant tumour in the world and second in cancer-related mortality. In 2018, there were 1,096,601 new cases and 551,269 deaths [1]. The incidence of CRC in China is showing a younger trend [2]. The decline in the quality of life of patients and the huge medical expenses pose a threat to human health and social development. Although the diagnosis and treatment of CRC have been greatly improved in recent years, the 5-year survival rate of patients has not been significantly improved [3], so it is urgent to improve the understanding of the exact mechanism of the disease and develop new targeted therapies.

MicroRNAs (miRNAs) are short noncoding RNAs with a length of approximately 22 nucleotides. At present, nearly 3000 such molecules have been discovered and are still being explored. miRNAs participate in the regulation of posttranscriptional gene expression by binding to the 3′-UTR of target mRNAs, leading to mRNA degradation or preventing its translation. A miRNA can target and regulate numerous mRNAs, and a target mRNA can also be regulated by multiple miRNAs, which have a complex network of relationships and play an important role in almost all biological pathways and regulate the occurrence and development of many human diseases, including cancer [4]. In recent decades, research on miRNAs has been in full swing, especially research on miRNAs in the occurrence and development of tumours. Studies have shown that miRNAs are dysregulated in a variety of cancers, and dysregulated miRNAs play a role in tumour cell proliferation, apoptosis, invasion and drug resistance and act as cancer-promoting genes or suppressor genes [5]. According to previous studies, some scholars summarized hundreds of miRNAs with high or low expression in lung cancer, gastric cancer, breast cancer, liver cancer and so on as well as the relevant mechanisms and pathways that affect the malignant progression of tumours and discussed the use of these miRNAs as the corresponding cancer diagnosis and treatment targets and prognostic indicators [6–9]. There are also many reports about miRNAs in CRC. Xu et al. reported the relationship between miRNA expression and tumour mutation burden in CRC [10]. miR-4319 can inhibit the proliferation, migration and invasion of CRC cells and inhibit the cell cycle, acting as a tumour suppressor gene in CRC by targeting ABTB1 [11]. MiR-629-5p is highly expressed in CRC tissues and cell lines, promotes the proliferation and migration of CRC cells, reduces the proportion of apoptotic cells by targeting CXXC4 and thus promotes the malignant progression of CRC [12]. These results suggest that differentially expressed miRNAs could be potential therapeutic targets for CRC. Differentially expressed miR-495-3p has been reported in various types of tumours, such as oesophageal cancer [13], osteosarcoma [14] and melanoma [15]. Eun et al. reported that miR-495-3p plays a tumour suppressive role in the development of gastric cancer by regulating a variety of epigenetic modifications [16]. miR-495-3p is regulated by the upstream molecules NEAT1 and LUNAR1 in CRC and plays a bridge role in the promotion of lncRNA in the development of CRC [17, 18]. However, there are few studies on the differential expression of miR-495-3p in CRC, and at the same time, whether miR-495-3p can regulate cell proliferation in CRC and the related mechanism of action are still unclear.

High mobility group box-1 (HMGB1) is a multifunctional nonhistone protein comprising 215 amino acids that are mainly distributed in the nucleus and play a key role in nucleosome structure and homeostasis, gene transcription, DNA recombination and damage repair, and maintenance of chromosome stability and telomere homeostasis. Studies have shown that HMGB1 plays an important role in promoting or suppressing tumorigenesis in various cancers, including CRC, and is associated with prognosis [19, 20]. We learned from the database that miR-495-3p had a complementary sequence with the 3′-UTR end of HMGB1 mRNA. Therefore, we hypothesized that there is a close relationship between HMGB1 and miR-495-3p that thus influences the behaviour of CRC cells.

In this study, we explored the role of miR-495-3p in CRC and its molecular mechanism, hoping to develop new targets for the diagnosis and treatment of CRC. We found that miR-495-3p was significantly underexpressed in CRC tissues and cell lines. MiR-495-3p inhibited the proliferation and migration and promoted the apoptosis of CRC cells by downregulating the expression of HMGB1, suggesting that miR-495-3p could be a potential target for the treatment of CRC.

## Materials and methods

### Acquisition of carcinoma and paracancerous tissue

CRC tissues and their paracancerous tissues were collected from 45 patients with colorectal cancer who had not undergone chemoradiotherapy at Yijishan Hospital. The tumour tissue and the surrounding normal tissue within 0.5 cm were collected during resection and stored in liquid nitrogen immediately, with the informed consent of the patient and the approval of the medical committee and the ethics committee of Yijishan Hospital.

### Cell culture

Four human CRC cell lines, HT29, SW480, SW620 and HCT116 and the human normal colon epithelial cell line FHC were purchased from Shanghai Cell Bank, Chinese Academy of Sciences (Shanghai, China). The complete cell medium contained 10% FBS (Gibco, Waltham, USA) and RPMI-1640 (Gibco, Carlsbad, USA) or DMEM (HyClone, Logan, USA). Four colorectal cancer cell lines were cultured in a complete RPMI-1640 medium, while normal colon epithelial FHC cells were cultured in DMEM. The environment of the cell incubator was kept at a constant temperature of 37 °C with 5% carbon dioxide. The cells were given fluid exchange or passage for 1–2 days.

### RNA extraction and reverse transcription-quantitative polymerase chain reaction (RT-qPCR)

RNA of tissues and cells was extracted using TRIzol (Invitrogen, Carlsbad, USA) according to the manufacturer’s step-by-step instructions. A Nanodrop 2000 Spectrophotometer (Thermo Fisher, Waltham, USA) was used to measure RNA concentration, and then 2 ml was taken for reverse transcription using a reverse transcription kit (Takara, Dalian, China) to obtain the corresponding cDNA. For miR-495-3P, we used the stem–loop RT primer Bulge-Loop hsa-miR-495-3p-RT Primer-2 (RiboBio, miR8000402) and Bulge-Loop U6-RT Primer (RiboBio, ssD0904071008). Real-time fluorescence quantitative PCR was performed using a qPCR kit (Takara, Dalian, China). The sequence of primers used in qPCR was as follows: U6, CTCGCTTCGGCAGCACA-forward and AACGCTTCACGAATTTGCGT-reverse; GAPDH, GAACGGGAAGCTCACTGG-forward and GCCTGCTTCACCACCTTCT-reverse; miR-495-3p, AAACAAACAUGGUGCACUUCUU-forward and GAAGUGCACCAUGUUUGUUUUU-reverse; and HMGB1, TGCTGATTAGTTACCACAGTTCTGA-forward and CTCGGGTACACAGGACACACAA-reverse. The primers above were all purchased from Ribo Biology (Guangzhou, China), and U6 was used as the internal reference for miR-495-3p while GAPDH was used as the internal reference for HMGB1. The expression of miR-495-3p and HMGB1 in tissues or cells was analysed by the 2−ΔΔ CT method, and the value of paracancerous tissues was normalized to 1.

### Cell transfection

MiR-495-3p mimics, inhibitors and their respective negative controls (control mimic and control inhibitor), the short hairpin RNA (shRNA) HMGB1 and negative control (sh-NC) were synthesized by Ribo Biology (Guangzhou, China). The HMGB1 overexpression plasmid and the empty plasmid were supplied by GenePharma Co., Ltd. (Shanghai, China). The original medium was replaced with serum-free medium Opti-MEM (Gibco) when the confluence degree of cells in the six-well plate reached 60–70%. Then, a Lipofectamine 3000 Kit (Invitrogen) was used for transfection according to the manufacturer’s instructions. After 6 h, the Opti-MEM medium was replaced with a medium containing 2% FBS or complete medium. Forty-eight hours after transfection, cells were collected for subsequent experiments.

### Cell survival test (CCK-8 assay)

We used a Cell Counting Kit-8 Kit (Keygen Biotech Co., Ltd., Nanjing, China) to analyse the viability of cells according to the instructions. After the corresponding transfection treatment, HT29 and SW480 cells were seeded into 96-well plates at 1 × 10^4^ cells per well. Ten microlitres of CCK-8 reagent was added to each well at 12, 24, 36, 48 and 60 h after transfection. The cells were returned to the incubator for further incubation for 2 h, and the absorbance was measured at 450 nm.

### Cell proliferation test (EDU assay)

Six hours after transfection, the cells were inoculated into 24-well plates. When the cells grew to 70–80% confluence, EdU reagent was added to each well at a ratio of 1:1000 and incubated for 2 h. Then, the cells were fixed and stained according to the manufacturer’s instructions (EDU Cell Proliferation Kit, Ribo, Guangzhou, China). Finally, fluorescence microscopy was used to observe the cells and image them.

### Cell migration test (Transwell assay)

Twenty-four hours after transfection, the cells were digested, and the concentration of cells was adjusted to 1 × 10^5^ cells/ml with serum-free RPMI-1640. A 100-μl cell suspension was added to the upper compartment of the Transwell chamber, while RPMI-1640 medium containing 20% serum was added to the lower compartment. After incubation for 48 h, the transmembrane cells were fixed with 4% paraformaldehyde and stained with 0.1% crystal violet, and the nontransmembrane cells were lightly wiped off with a cotton swab. Finally, an inverted microscope was used for observation and imaging.

### Cell apoptosis test (flow cytometry)

To detect apoptosis rates, we purchased apoptosis detection kits (BD Biosciences, CA, USA). Forty-eight hours after transfection, the cells were collected, washed with PBS three times and resuspended in an appropriate amount of binding buffer. Then, 1 μl annexin-V–fluorescein isothiocyanate (FITC) was added for 15 min, and 1 μl propidium iodide (PI) was added. (Each cell line was set with FITC staining alone, PI staining alone and blank control to adjust the drawing gate.) Finally, apoptosis was detected by flow cytometry.

### Correlation between miR-495-3p and HMGB1 (dual-luciferase reporter gene assay)

To evaluate the direct binding of miR-495-3p to HMGB1, we used a dual-luciferase reporter assay. After the wild-type and mutant vectors of the HMGB1 3′-UTR were designed, the miR-495-3p mimic or control mimic and wild-type or mutant-type vector plasmid were cotransfected into 293T cells (a very common cell line expressing foreign genes for study, which is relatively easy to transfect). The cotransfection groups were as follows: miR-495-3p mimic + MUT, miR-495-3p mimic + WT, control mimic + MUT and control mimic + WT. After 24 h, a dual-luciferase reporter assay kit was used for experimental analysis according to the instructions.

### In vivo experiment of nude mice (armpit tumour formation)

We purchased 3-week-old SPF (special-solution-free) grade male nude mice from Hangzhou Ziyuan Laboratory Animal Science and Technology Co., Ltd. (Hangzhou, China). A 1-week quarantine was conducted in the quarantine room of the SPF animal laboratory of Wannan Medical College. Approximately 2 × 10^6^ HT29 cells were injected subcutaneously into the left axilla of each nude mouse. After that, the tumour volume was observed and measured every 4 days. After 20 days, the mice were sacrificed with cervical dislocation, and the tumour bodies were isolated and weighed. All operations passed the experimental animal welfare and ethics review of Wannan Medical College.

### Protein extraction and Western blotting

After 48 h of transfection, the culture medium was removed and discarded. Two hundred microlitres of radioimmunoprecipitation (RIPA) lysis buffer (Thermo Fisher, MA, USA) containing protease inhibitor was added to each well of the six-well plate, and then the protein in colorectal cancer cells was dissolved at 4 °C for 30 min. Then, the supernatant containing protein was centrifuged at 4 °C under 7500 centrifugal force for 5 min. Proteins were isolated by SDS-PAGE constant pressure 80–120 V electrophoresis and then transferred to PVDF membranes at a constant current of 300 mA. After sealing at room temperature for 1 h, the required HMGB1 (24 kDa) and internal reference β-actin (42 kDa) protein bands were cut and placed into a 1:1000 ratio of primary antibody (ABclonal, Woburn, USA) reaction solution overnight incubation at 4 °C. The next day, after washing the membrane with TBST three times, the membrane was incubated in 1:5000 secondary antibody solution at room temperature for 2 h. An exposure analysis was performed using a luminescent solution after washing the membrane again. The ImageJ software was used to process the grey values of the strips.

### Data analysis method

According to the mean and standard deviation of the three independent repeated experiments, the GraphPad Prism-8 software was used for analysis and mapping. The corresponding *P* value was calculated by a *t* test. *P* < 0.05 indicated a significant difference and is represented by *, while *P* < 0.01 is denoted by ** and *P* < 0.001 is denoted by ***.

## Results

### miR-495-3p expression is decreased in CRC tissues and cell lines

**Fig. 1:**
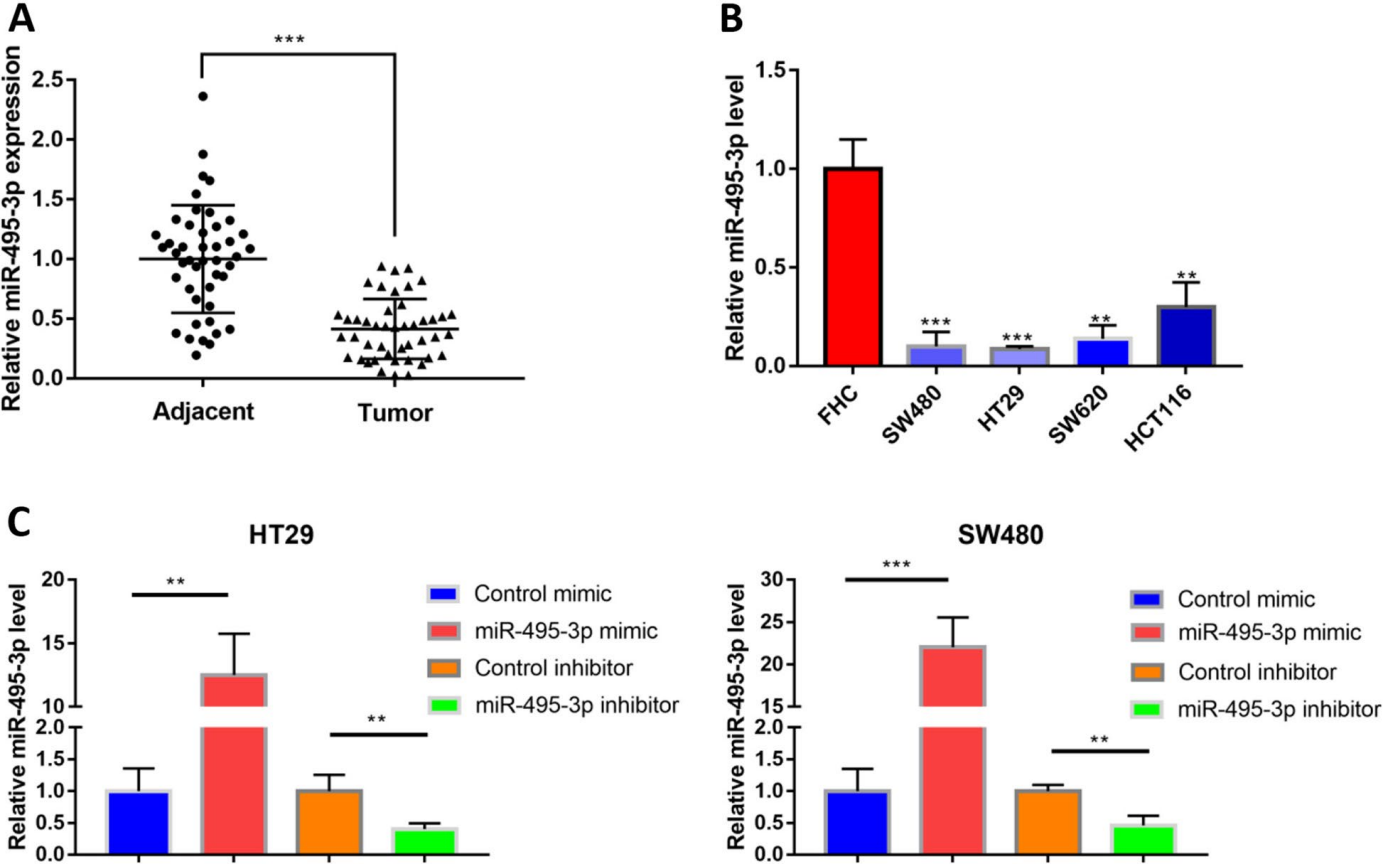
miR-495-3p is underexpressed in colorectal cancer. **A** miR-495-3p levels in 45 pairs of CRC tissues and their paracancer tissues assessed by qRT-PCR. **B** miR-495-3p levels in HT29, SW480, SW620, HCT116 and FHC cells assessed by qRT-PCR. **C** miR-495-3p levels in HT29 and sw480 cells transfected with the control mimic, miR-495-3p mimic, control inhibitor or miR-495-3p inhibitor assessed by qRT-PCR. qRT-PCR, real-time quantitative polymerase chain reaction. *p < 0.05, **p < 0.01 and ***p < 0.001

We used RT-qPCR to detect the expression level of miR-495-3p in 45 pairs of CRC tissues and their paracancerous tissues, four types of CRC cells (HT29, SW480, SW620 and HCT116) and one normal colon epithelial cell line (FHC). The results showed that the expression of miR-495-3p was significantly lower in CRC tissues than in the corresponding paracancerous tissues (Fig. 1A). Compared with normal intestinal epithelial cells, the level of miR-495-3p in CRC cells was also prominently reduced (Fig. 1B), and the differential expression of HT29 and SW480 cells was the most significant. Therefore, these two cell lines were selected to complete the following experiment. To further understand the specific role of miR-495-3p in CRC, we transfected miR-495-3p mimic, control mimic, miR-495-3p inhibitor and control inhibitor into HT29 and SW480 cells to complete the high and low expression of miR-495-3p, respectively. The RT-qPCR results confirmed that this step was successful (Fig. 1C). At this point, we learned that miR-495-3p was underexpressed in CRC tissues and cell lines.

### miR-495-3p inhibits proliferation and migration and promotes the apoptosis of CRC cells

**Fig. 2:**
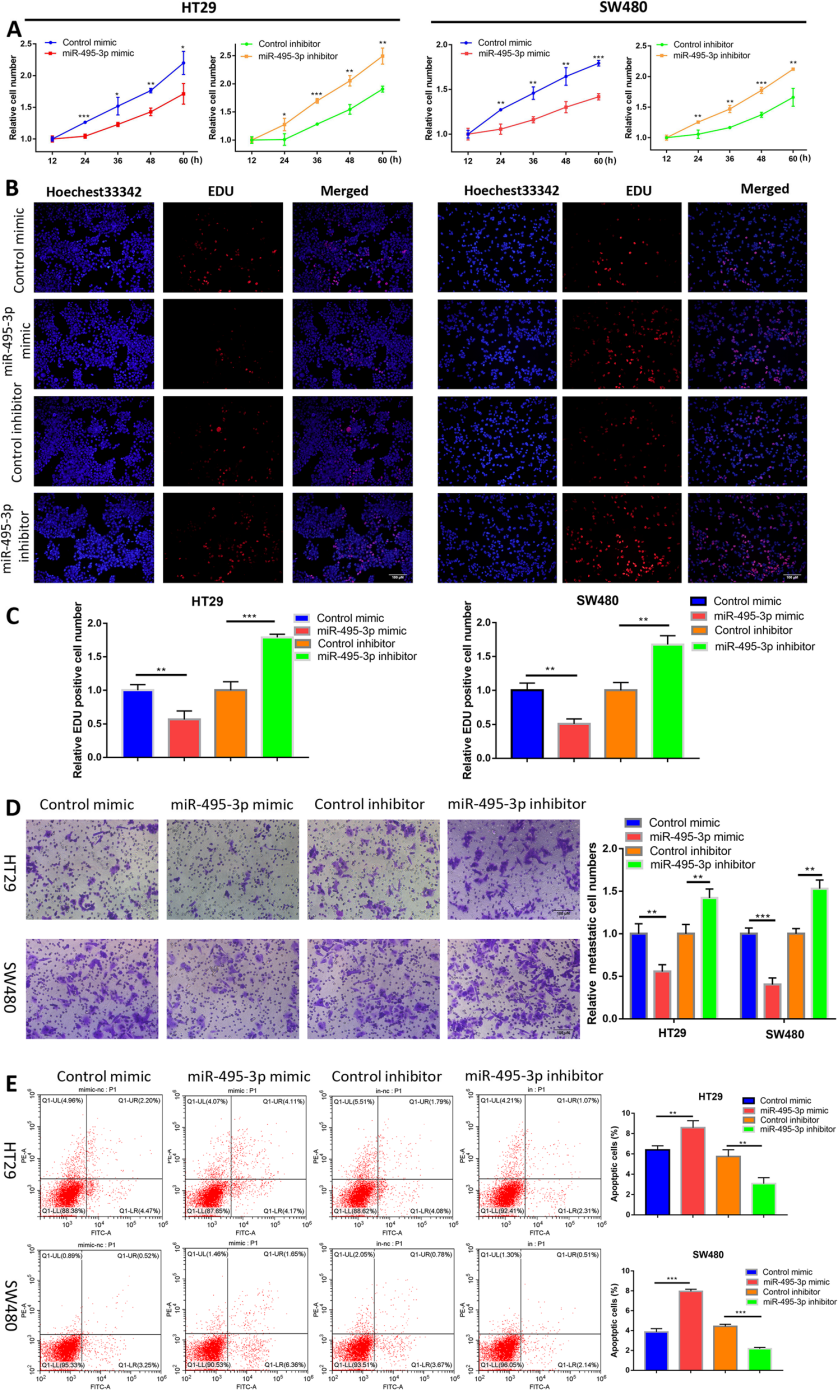
miR-495-3p suppresses the malignant phenotypes of CRC cells. **A** Relative CRC cell growth rates at 12 h, 24 h, 36 h, 48 h and 60 h after knockdown or upregulation of miR-495-3p were measured using a CCK-8 assay. **B** The effect of miR-495-3p on CRC cell proliferation was assessed by an Edu assay. **C** Relative Edu-positive cell numbers after miR-495-3p knockdown and overexpression. **D** Transwell assays were performed to evaluate cellular migration after miR-495-3p knockdown and overexpression. **E** Flow cytometry apoptosis experiments were used to measure the apoptosis rate of HT-29 and SW480 cells after miR-495-3p knockdown and overexpression. *p < 0.05, **p < 0.01 and ***p < 0.001

To investigate the effect of miR-495-3p on the proliferation of colorectal cancer cells, we performed CCK-8 and EdU experiments on CRC cells (HT29 and SW480) transfected with miR-495-3p mimic and inhibitor and their respective control groups. The results showed that compared with the control group, the overexpression of miR-495-3p significantly reduced the proliferation ability of cells, while the proliferation ability of cells in the low-expression group of miR-495-3p was increased (Fig. 2A–C). Meanwhile, the Transwell assay was used to explore the effect of miR-495-3p on the migration of CRC cells. As shown in the figures, fewer migrated cells were found in the group with increased miR-495-3p expression compared with the control group, while more migrated cells were found in the group with decreased miR-495-3p expression compared with the control group (Fig. 2D). The next experiment was to detect the influence of miR-495-3p on the apoptosis of CRC cells by flow cytometry. As we suspected, the proportion of apoptotic cells after overexpression of miR-495-3p was increased, while the proportion was decreased after knockdown of miR-495-3p compared with the respective control group (Fig. 2E). In general, we proved that miR-495-3p could inhibit the proliferation and migration of CRC cells and promote cell apoptosis, while knockdown of miR-495-3p could achieve the opposite result.

### HMGB1 is the target gene of miR-495-3p, with abundant expression in CRC

**Fig. 3:**
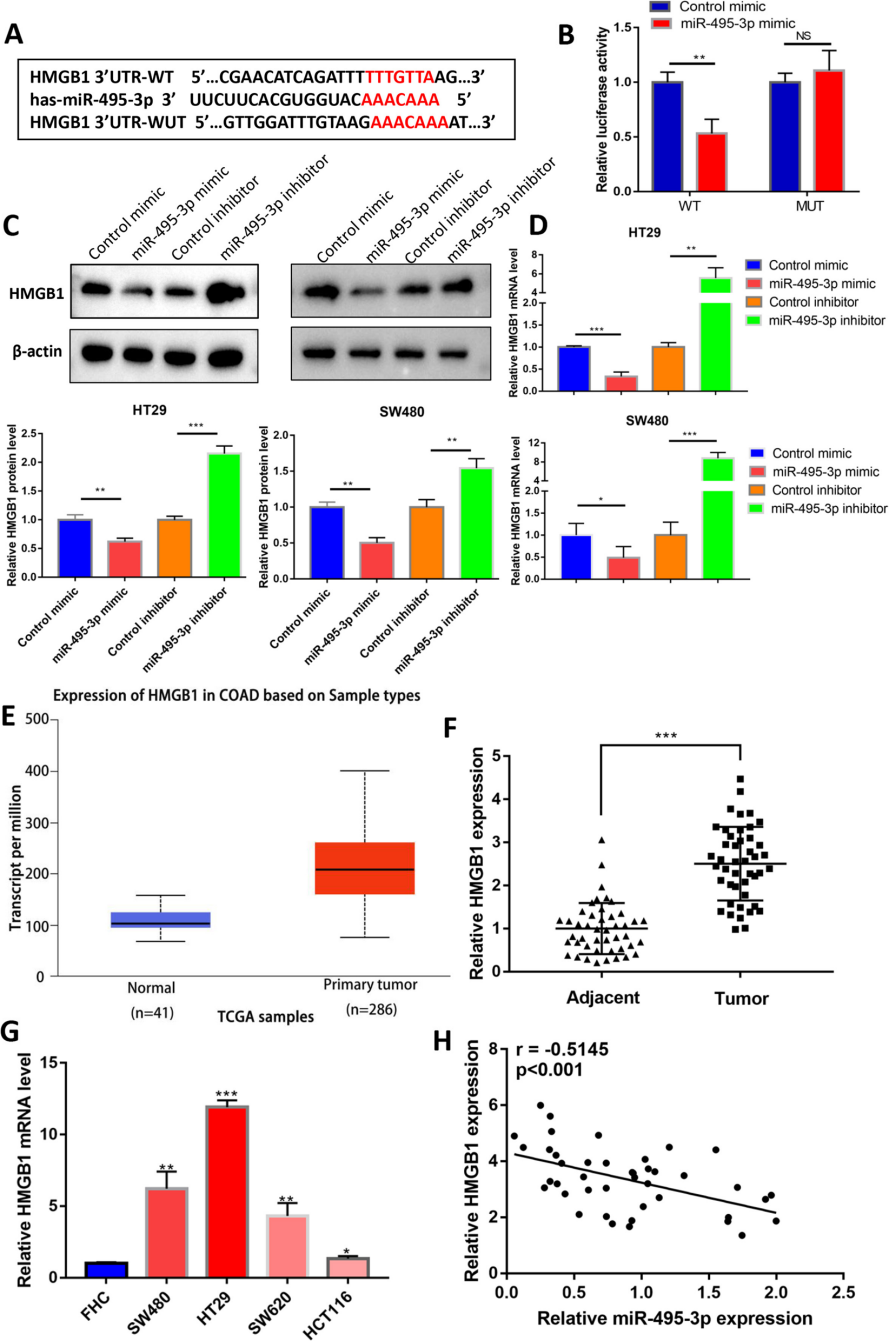
HMGB1 is overexpressed in CRC and is the target gene of miR-495-3p. **A** Target genes of miR-495-3p were predicted using TargetScan and RNAhybrid. **B** The relative luciferase activity was measured by luciferase reporter assay. **C** Western blotting for HMGB1 protein levels following miR-495-3p mimic/inhibitor transfection. **D** HMGB1 mRNA levels in HT29 and SW480 cells after miR-495-3p knockdown and overexpression assessed by qRT-PCR. **E** TCGA database analysis of HMGB1 expression in CRC. **F** HMGB1 mRNA levels in 45 pairs of CRC tissues and their paracancer tissues assessed by qRT-PCR. **G** HMGB1 mRNA levels in HT29, SW480, SW620, HCT116 and FHC cells assessed by qRT-PCR. **H** Negative correlation between miR-495-3p and HMGB1 expression in 45 paired colorectal cancer specimens. *p < 0.05, **p < 0.01 and ***p < 0.001

**Fig. 4:**
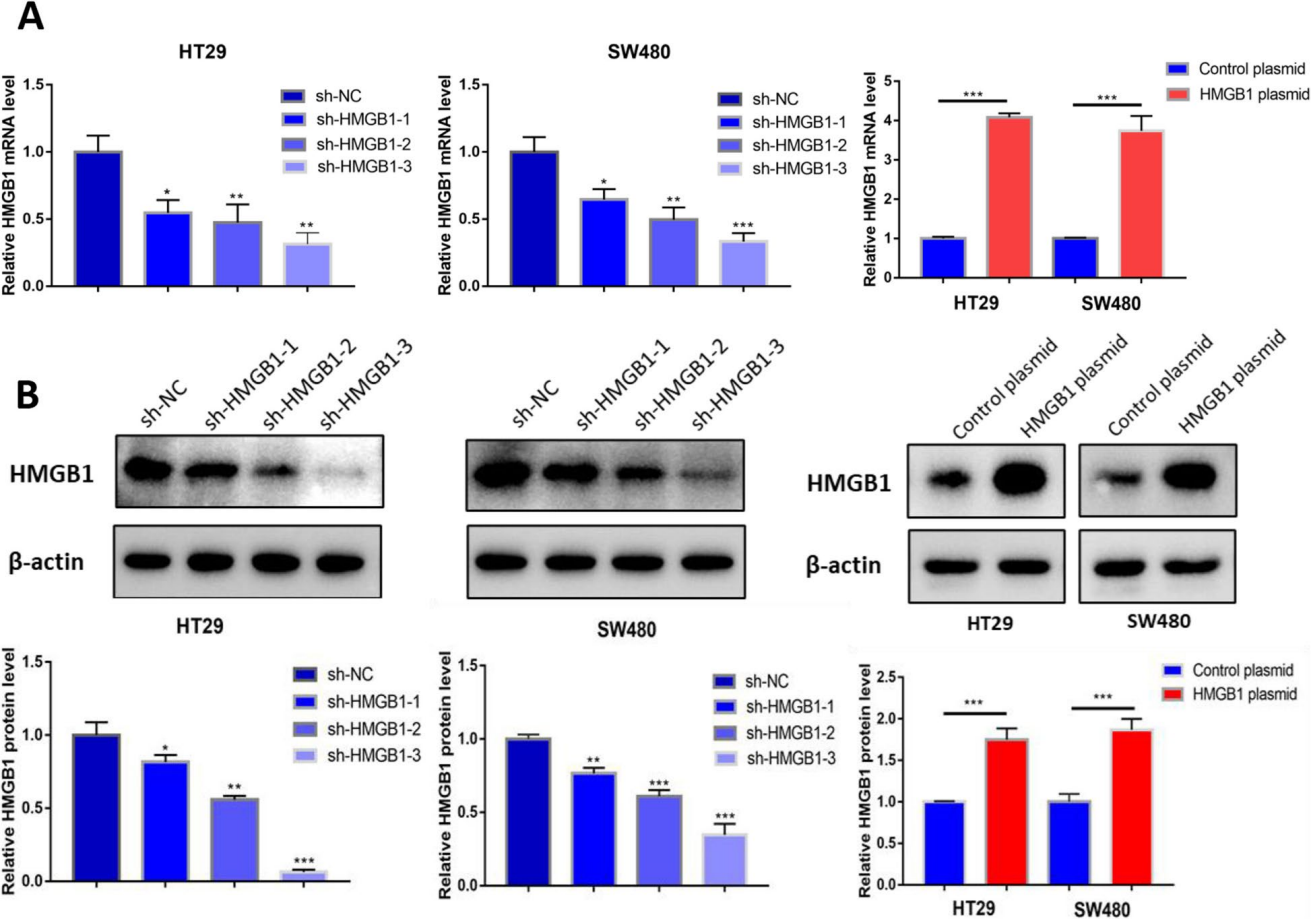
**A** The efficiency of HMGB1 knocking down and overexpressing was assessed by qPCR. **B** The efficiency of HMGB1 knocking down and overexpressing was assessed by Western blotting. *p < 0.05, **p < 0.01 and ***p < 0.001

To date, we have confirmed that miR-495-3p can inhibit the malignant biological behaviour of CRC cells. To explore the mechanism of miR-495-3p’s action more specifically, we used two databases (TargetScan and RNAhybrid) to predict the downstream target genes of miR-495-3p. We discovered complementary sequences in the 3′-UTR of HMGB1 mRNA and miR-495-3p, which may be the direct binding site of the two molecules (Fig. 3A). To verify this hypothesis, we constructed HMGB1 wild-type and mutant-type luciferase reporter gene plasmids and then cotransfected these plasmids into 293T cells with miR-495-3p mimic and control mimic. In the HMGB1 3′-UTR wild type, luciferase activity was distinctly reduced after overexpression of miR-495-3p compared with the control group. However, in the mutants whose binding sequences were changed, there was no significant difference in luciferase activity between the miR-495-3p overexpression group and the control group (Fig. 3B). After transfection with miR-495-3p overexpression, knockdown and corresponding controls, we detected the expression of HMGB1 protein by Western blotting and HMGB1 mRNA by qPCR. As expected, compared with the control group, the protein and mRNA levels of HMGB1 decreased after overexpression of miR-495-3p, while the protein and mRNA levels of HMGB1 increased after reducing the expression of miR-495-3p (Fig. 3C, D). To go a step further, HMGB1 is the target of miR-495-3p. After that, we found that the expression of HMGB1 was upregulated in CRC using the TCGA database (Fig. 3E). Moreover, the qPCR results showed that the HMGB1 levels in CRC tissues were significantly higher than those in adjacent tissues (Fig. 3F). Compared with FHC, the level of HMGB1 in four CRC cell lines was significantly increased. Among them, HT29 and SW480 cells showed the most obvious differential expression, which was consistent with the cell lines with the most significant differential expression of miR-495-3p (Fig. 3G), indicating that HMGB1 was highly expressed in CRC and had a certain negative correlation with the expression of miR-495-3p (Fig. 3H). Overall, HMGB1, which is highly expressed in CRC, is the target gene of miR-495-3p. To further study the orientation that HMGB1 contributes to CRC progression, three sh-RNAs and an HMGB1 overexpression plasmid were constructed. Figure 4 shows the knockdown and overexpression efficiency of HMGB1. Among the three sh-HMGB1 shRNAs, the knockdown efficiency of sh-HMGB1-3 was the highest, so we chose sh-HMGB1-3 to complete the knockdown intervention step.

### HMGB1 promotes the proliferation and migration of CRC cells and inhibits apoptosis

**Fig. 5:**
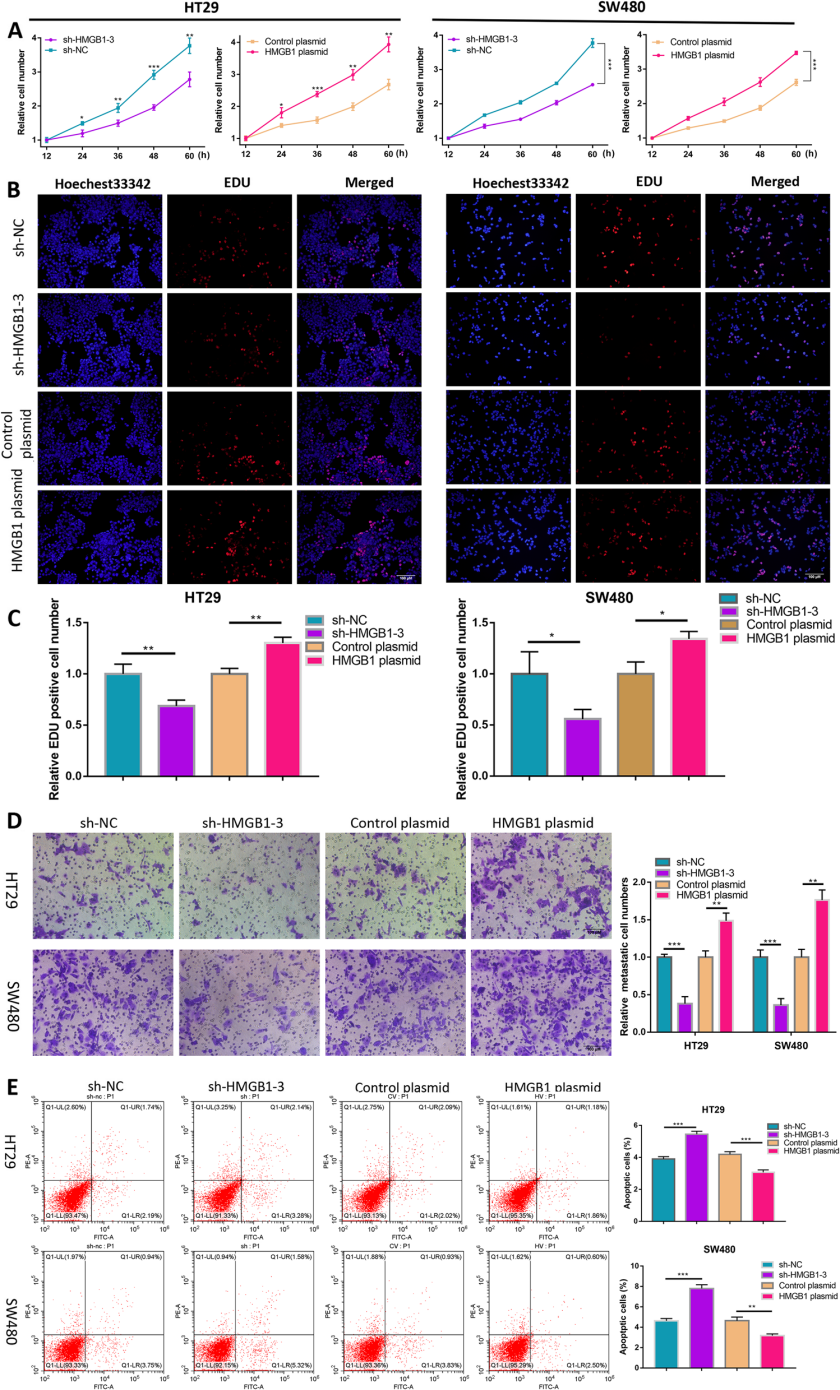
HMGB1 facilitates malignant phenotypes of CRC cells. **A** Relative CRC cell growth rates at 12 h, 24 h, 36 h, 48 h and 60 h after knockdown or upregulation of HMGB1 were measured using a CCK-8 assay. **B** The effect of HMGB1 on CRC cell proliferation was assessed by an Edu assay. **C** Relative Edu-positive cell numbers after HMGB1 knockdown and overexpression. **D** Transwell assays were performed to evaluate cellular migration after HMGB1 knockdown and overexpression. **E** Flow cytometry apoptosis experiments were used to measure the apoptosis rate of HT-29 and SW480 cells after HMGB1 knockdown and overexpression. *p < 0.05, **p < 0.01 and ***p < 0.001

After HMGB1 knockdown and overexpression, cell proliferation was also detected by CCK-8 and EdU assays. Transwell assays were used to detect cell migration, and cell apoptosis was detected by flow cytometry. The CCK-8 and EdU assays revealed that, compared with the control group, the proliferation ability of cells in the HMGB1 knockdown group was decreased, while the proliferation ability was increased in the overexpression group (Fig. 5A–C). The Transwell results indicated that reduced HMGB1 expression resulted in reduced migratory cells, while higher HMGB1 expression resulted in a corresponding increase in migratory cells (Fig. 5D). The subsequent flow cytometry showed that knockdown of HMGB1 inhibited apoptosis, while overexpression of HMGB1 had the opposite effect (Fig. 5E). In short, HMGB1 promotes proliferation and migration and inhibits apoptosis of CRC cells.

### miR-495-3p inhibits the proliferation and migration of CRC by targeting HMGB1 in vivo and in vitro

**Fig. 6:**
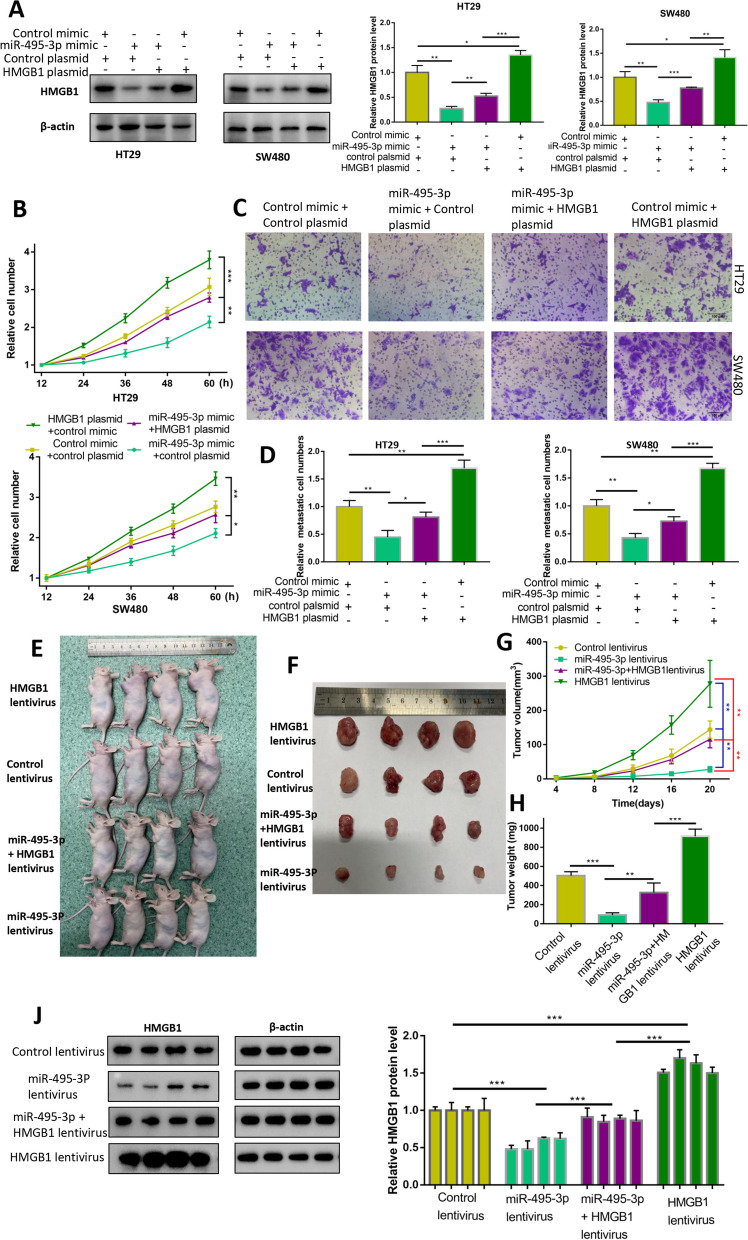
miR-495-3p exerted its biological function by targeting HMGB1 in vivo and in vitro. **a** HMGB1 protein levels were restored after co-transfection with miR-495-3p mimics and HMGB1 plasmid in HT29 and SW480 cells. **B** Overexpression of HMGB1 attenuated the effect of miR-495-3p on the proliferation of CRC cells. **C**, **D** Overexpression of HMGB1 attenuated the effect of miR-495-3p on the migration of CRC cells. **E**, **F** Representative images of implanted mice and dissected tumour tissues. The HMGB1 lentivirus group with the largest tumour volume was placed at the front, and the miR-495-3p lentivirus group with the smallest tumour volume was placed at the end for the sake of facilitating the observation of tumour size. **G** Growth curves of the tumours. Tumour volumes were measured using a slide calliper every 4 days (*n* = 4). **H** Weights of xenograft tumour in different groups (*n* = 4). **J** HMGB1 protein levels in xenograft tumour tissues were detected by Western blot analysis. *p < 0.05, **p < 0.01 and ***p < 0.001

Rescue experiments were conducted to further verify the function of miR-495-3p by targeting HMGB1. Western blotting showed that the protein level of HMGB1 decreased significantly after overexpression of miR-495-3p alone and increased correspondingly after overexpression of HMGB1 alone, while the protein level of HMGB1 was between the two when miR-495-3p and HMGB1 were overexpressed at the same time, indicating that cotransfection of miR-495-3p mimic and HMGB1 overexpression plasmid could weaken the negative regulation of HMGB1 by miR-495-3p (Fig. 6A). Further functional experiments with CCK-8 showed that overexpression of HMGB1 attenuated the inhibitory effect of miR-495-3p on the proliferation of CRC cells compared with negative controls (Fig. 6B). The Transwell experiments also confirmed that the inhibitory effect of miR-495-3p on the migration of CRC cells was weakened when HMGB1 and miR-495-3p were overexpressed synchronously (Fig. 6C, D). These results suggest that miR-495-3p can inhibit the proliferation and migration of CRC cells by targeting HMGB1 in vitro. To elucidate the impact and mechanism of miR-495-3p on tumour formation in vivo, HT29 cells stably transfected with miR-495-3p overexpression or/and HMGB1 overexpression were inoculated subcutaneously into the left armpit of nude mice (Fig. 6E). According to the experiment, compared with the control lentivirus group, the tumour weight and volume in the miR-495-3p lentivirus group were significantly decreased, and the tumour weight and volume in the HMGB1 lentivirus group were significantly increased. However, the tumour weight and volume of the miR-495-3p and HMGB1 lentivirus group were between those of the former two groups (Fig. 6F–H). The Western blotting results of tumour tissues displayed the lowest expression of HMGB1 protein in the miR-495-3p lentivirus group and the highest in the HMGB1 lentivirus group. Similarly, the HMGB1 protein level of the miR-495-3p and HMGB1 co-overexpression group was located between that of the miR-495-3p overexpression group and the HMGB1 overexpression group (Fig. 6J). These results indicate that miR-495-3p can inhibit colorectal tumour growth in vivo while HMGB1 promotes tumour growth and further indicate that miR-495-3p can inhibit CRC cell proliferation by targeting HMGB1.

### Conclusion

In summary, our research shows that miR-495-3p inhibits the progression of colorectal cancer by downregulating the expression of HMGB1 in vivo and in vitro, which indicates that miR-495-3p may become a potential therapeutic target for colorectal cancer.

## Discussion

miR-495-3p is involved in many pathophysiological processes. A study verified that miR-495-3p can play a role in intervertebral disc degeneration (IVDD) by inhibiting inflammation and apoptosis of human nucleus pulposus cells by targeting IL5RA [21]. Another study also suggested that miR-495-3p may be related to lung function and chronic obstructive pulmonary emphysema (COPD) [22]. miR-495-3p has been extensively studied in cancer, including the occurrence, development and drug resistance of cancer. A study clarified that functional loss or inhibition of miR-495-3p can trigger the overexpression of a variety of oncogenic epigenetic modifiers (EMS), thereby promoting malignant transformation and growth of gastric epithelial cells [16]. Chen et al. found that miR-495-3p inhibited multidrug resistance in gastric cancer by regulating autophagy through the GRP78/mTOR axis [23]. There are also reports that miR-495-3p targets TGFβR1, TGFβR2, SMAD4 and BUB1 [24]. To the best of our knowledge, few researchers have reported the specific role and mechanism of miR-495-3p and its relationship with HMGB1 in CRC. In this study, we found that the expression of miR-495-3p was decreased in CRC tissues and cells, and artificial intervention of miR-495-3p expression affected the malignant phenotype of CRC cells. After the overexpression of miR-495-3p, the proliferation and migration ability of CRC cells decreased, and the proportion of apoptosis increased. Conversely, miR-495-3p knockdown increased the proliferation and migration ability and reduced the proportion of apoptosis. These results suggest that miR-495-3p may play a role as a tumour suppressor gene in CRC.

To further explore the potential mechanism of miR-495-3p in CRC, we concluded that HMGB1 might be the target of miR-495-3p through a bioinformatics analysis. HMGB1 consists of 215 amino acids with two DNA-binding domains (box A and box B) and an acidic tail. Among them, box A plays an antagonistic role, while box B plays an A cytokine-inducing role. HMGB1 binds to DNA through the DNA-binding domain and regulates the structure of chromosomes, thereby regulating the gene transcription [25]. Overexpression and abnormal secretion of HMGB1 play an important role in many diseases. Previous studies have shown that HMGB1 plays a role in sepsis, atherosclerosis, rheumatoid arthritis and other inflammation-related diseases [26]. In recent years, many studies have focused on its role in a variety of cancers, including CRC. Various reports have shown that HMGB1 expression is significantly upregulated in breast cancer, gastric cancer, lung cancer and other cancers and downregulated in pancreatic cancer [27–30]. Researchers have reported that HMGB1 may regulate the reactivity of rectal cancer cells to preoperative chemoradiotherapy [31]. Ueda et al. examined the expression of HMGB1 in cancer tissues and normal paracancerous tissues of 140 patients with CRC using qPCR and found that the expression of HMGB1 in tumour tissues was significantly higher than that in normal tissues, and the high expression of HMGB1 was closely associated with a larger tumour volume, higher lymph node metastasis rate and lower survival rate [32]. In our research, we also confirmed the high expression of HMGB1 in CRC tissues and cells, and the high expression of HMGB1 can promote the proliferation and migration of CRC cells. We also revealed the negative regulatory relationship between HMGB1 and miR-495-3p and confirmed that HMGB1 was the downstream target of miR-495-3p through the dual-luciferase reporter gene assay. We also found that the expression of HMGB1 protein and mRNA was significantly decreased after the upregulation of miR-495-3p. Conversely, the HMGB1 protein and mRNA levels were significantly increased after the downregulation of miR-495-3p. Further rescue experiments showed that, compared with the control group, HMGB1 could reduce the inhibitory effect of overexpression of miR-495-3p on the proliferation and migration of CRC cells, and this consequence was also verified in vivo by our tumour formation experiment in nude mice. These results suggest that miR-495-3p plays a role as a tumour suppressor gene in CRC by targeting HMGB1 in vivo and in vitro. In fact, not only miRNAs but also lncRNAs and circRNAs play a crucial role in tumorigenesis and development, and differentially expressed lncRNAs and circRNAs in CRC can serve as potential therapeutic targets for CRC [33]. There are often complicated relationships when they work. For example, linc01224 targets the miR-485-5p/MYO6 axis to promote CRC progression [34], circ_0082182 functions as an oncogene in CRC by sponging miR-411 or miR-1205 to activate the Wnt/β-catenin pathway [35], and the circ0009910/miR-145-5p/PEAK1 axis contributes to the pathogenesis of CRC [36]. Our miR-495-3P may also be regulated by lncRNA or circRNA, which may become the content of our future research.

## Conclusions

In conclusion, we confirmed the low expression of miR-495-3p in CRC and high expression of HMGB1 in CRC. Moreover, miR-495-3p regulates the proliferation, migration and apoptosis of CRC cells by targeting HMGB1 in vivo and in vitro, revealing the expectation that miR-495-3p could be used as a potential therapeutic target for CRC. In other words, we discovered a new regulatory network that affects CRC, and the upstream molecular and downstream pathways of this regulatory pattern may be studied in detail in the future.

## Data Availability

All authors ensure that our data does not contain any of the following: (a) Infringes or makes unauthorized use of the Intellectual Property Rights. (b) Any other right of any person. (c) Is defamatory, derogatory, discriminatory or violates any rights of privacy. (d) Breaches any applicable law or regulation. (e) Contains a virus, malware or other potentially harmful components, information or instructions. (f) Is indecent, obscene or offensive. Please confirm agreement with this statement.

## References

[1] Bray F, Ferlay J, Soerjomataram I, Siegel RL, Torre LA, Jemal A (2018). Global cancer statistics 2018: GLOBOCAN estimates of incidence and mortality worldwide for 36 cancers in 185 countries. CA Cancer J Clin.

[2] Siegel RL, Miller KD, Fedewa SA (2017). Colorectal cancer statistics, 2017. CA Cancer J Clin.

[3] Dienstmann R, Salazar R, Tabernero J (2015). Personalizing colon cancer adjuvant therapy: selecting optimal treatments for individual patients. J Clin Oncol.

[4] Acunzo M, Romano G, Wernicke D, Croce CM (2015). MicroRNA and cancer--a brief overview. Adv Biol Regul.

[5] Iorio MV, Croce CM (2012). MicroRNA dysregulation in cancer: diagnostics, monitoring and therapeutics. A comprehensive review. EMBO Mol Med.

[6] Iqbal MA, Arora S, Prakasam G, Calin GA, Syed MA (2019). MicroRNA in lung cancer: role, mechanisms, pathways and therapeutic relevance. Mol Asp Med.

[7] Shin VY, Chu KM (2014). MiRNA as potential biomarkers and therapeutic targets for gastric cancer. World J Gastroenterol.

[8] Kandettu A, Radhakrishnan R, Chakrabarty S, Sriharikrishnaa S, Kabekkodu SP (2020). The emerging role of miRNA clusters in breast cancer progression. Biochim Biophys Acta Rev Cancer.

[9] Nagy Á, Lánczky A, Menyhárt O, Győrffy B (2018). Validation of miRNA prognostic power in hepatocellular carcinoma using expression data of independent datasets. Sci Rep.

[10] Xu L, Zheng Q (2021). Identification and validation of a miRNA-related expression signature for tumor mutational burden in colorectal cancer. World J Surg Oncol.

[11] Huang L, Zhang Y, Li Z, Zhao X, Xi Z, Chen H (2019). MiR-4319 suppresses colorectal cancer progression by targeting ABTB1. United European Gastroenterol J.

[12] Lu J, Lu S, Li J, Yu Q, Liu L, Li Q (2018). MiR-629-5p promotes colorectal cancer progression through targetting CXXC finger protein 4. Biosci Rep.

[13] Huang GM, Zang HL, Geng YX, Li YH (2020). LncRNA FAM83A-AS1 aggravates the malignant development of esophageal cancer by binding to miR-495-3p. Eur Rev Med Pharmacol Sci.

[14] Zhao G, Zhang L, Qian D, Sun Y, Liu W (2019). miR-495-3p inhibits the cell proliferation, invasion and migration of osteosarcoma by targeting C1q/TNF-related protein 3. Onco Targets Ther.

[15] Xia Y, Zhou Y, Han H, Li P, Wei W (2019). lncRNA NEAT1 facilitates melanoma cell proliferation, migration, and invasion via regulating miR-495-3p and E2F3. J Cell Physiol.

[16] Eun JW, Kim HS, Shen Q, Yang HD, Kim SY, Yoon JH (2018). MicroRNA-495-3p functions as a tumor suppressor by regulating multiple epigenetic modifiers in gastric carcinogenesis. J Pathol.

[17] He Z, Dang J, Song A, Cui X, Ma Z, Zhang Z (2019). NEAT1 promotes colon cancer progression through sponging miR-495-3p and activating CDK6 in vitro and in vivo. J Cell Physiol.

[18] Qian J, Garg A, Li F, Shen Q, Xiao K (2020). lncRNA LUNAR1 accelerates colorectal cancer progression by targeting the miR-495-3p/MYCBP axis. Int J Oncol.

[19] Kang R, Zhang Q, Zeh HJ, Lotze MT, Tang D (2013). HMGB1 in cancer: good, bad, or both?. Clin Cancer Res.

[20] Bao G, Qiao Q, Zhao H, He X (2010). Prognostic value of HMGB1 overexpression in resectable gastric adenocarcinomas. World J Surg Oncol.

[21] Lin X, Lin Q (2020). MiRNA-495-3p attenuates TNF-α induced apoptosis and inflammation in human nucleus pulposus cells by targeting IL5RA. Inflammation..

[22] Zhou T, Yu Q, Sun C, Wang Y, Zhong Y, Wang G (2018). A pilot study of blood microRNAs and lung function in young healthy adults with fine particulate matter exposure. J Thorac Dis.

[23] Chen S, Wu J, Jiao K, Wu Q, Ma J, Chen D (2018). MicroRNA-495-3p inhibits multidrug resistance by modulating autophagy through GRP78/mTOR axis in gastric cancer. Cell Death Dis.

[24] Zhou W, Guan W, Zhou Y, Rao Y, Ji X, Li J (2021). Weighted genes associated with the progression of retinoblastoma: evidence from bioinformatic analysis. Exp Eye Res.

[25] Cheng KJ, Alshawsh MA, Mejia Mohamed EH, Thavagnanam S, Sinniah A, Ibrahim ZA (2020). HMGB1: an overview of its versatile roles in the pathogenesis of colorectal cancer. Cell Oncol (Dordr).

[26] Yang H, Wang H, Chavan SS, Andersson U (2015). High mobility group box protein 1 (HMGB1): the prototypical endogenous danger molecule. Mol Med.

[27] Wang XH, Zhang SY, Shi M, Xu XP (2020). HMGB1 promotes the proliferation and metastasis of lung cancer by activating the Wnt/β-catenin pathway. Technol Cancer Res Treat.

[28] Wang CQ, Huang BF, Wang Y, Hu GR, Wang Q, Shao JK (2020). Expression of HMGB1 protein in breast cancer and its clinicopathological significance. Zhonghua Bing Li Xue Za Zhi.

[29] Fang J, Ge X, Xu W, Xie J, Qin Z, Shi L (2020). Bioinformatics analysis of the prognosis and biological significance of HMGB1, HMGB2, and HMGB3 in gastric cancer. J Cell Physiol.

[30] Kang R, Xie Y, Zhang Q, Hou W, Jiang Q, Zhu S (2017). Intracellular HMGB1 as a novel tumor suppressor of pancreatic cancer. Cell Res.

[31] Hongo K, Kazama S, Tsuno NH, Ishihara S, Sunami E, Kitayama J (2015). Immunohistochemical detection of high-mobility group box 1 correlates with resistance of preoperative chemoradiotherapy for lower rectal cancer: a retrospective study. World J Surg Oncol.

[32] Ueda M, Takahashi Y, Shinden Y, Sakimura S, Hirata H, Uchi R (2014). Prognostic significance of high mobility group box 1 (HMGB1) expression in patients with colorectal cancer. Anticancer Res.

[33] Zhu H, Yu J, Zhu H, Guo Y, Feng S (2017). Identification of key lncRNAs in colorectal cancer progression based on associated protein-protein interaction analysis. World J Surg Oncol.

[34] Gu J, Dong L, Wang Y, Nie W, Liu W, Zhao JA (2021). LINC01224 promotes colorectal cancer progression through targeting miR-485-5p/MYO6 axis. World J Surg Oncol.

[35] Liu R, Deng P, Zhang Y, Wang Y, Peng C (2021). Circ_0082182 promotes oncogenesis and metastasis of colorectal cancer in vitro and in vivo by sponging miR-411 and miR-1205 to activate the Wnt/β-catenin pathway. World J Surg Oncol.

[36] Kadkhoda S, Taslimi R, Noorbakhsh F, Darbeheshti F, Bazzaz JT, Ghafouri-Fard S (2021). Importance of Circ0009910 in colorectal cancer pathogenesis as a possible regulator of miR-145 and PEAK1. World J Surg Oncol.

