# Correction to: miR-495-3p depresses cell proliferation and migration by downregulating HMGB1 in colorectal cancer

**DOI:** 10.1186/s12957-022-02589-z

**Published:** 2022-04-30

**Authors:** Jie Ling Zhang, Hui Fen Zheng, Kai Li, Yi Ping Zhu

**Affiliations:** 1 Department of Oncology, The First Afliated Hospital of Wannan Medical College, Wuhu, 241002 China; 2 Department of Clinical Medicine, Wannan Medical College, Wuhu, 241002 China; 3 Anhui Province Key Laboratory of Biological Macro-molecules Research, Wannan Medical College, Wuhu, 241002 China

**Correction to: World J Surg Oncol 20, 101 (2022)**

**https://doi.org/10.1186/s12957-022-02500-w**

Following the publication of the original article [1], it has been reported that there are errors in the article.

1. In figure 2, the pictures of control mimic group and miR-495-3p mimic group are reversed in SW480 cell line.

2. In Materials and methods (qRT-PCR), the "and then 2 ml was taken for..." should be "and then 2 microlitres was taken for...".

3. In Result section (HMGB1 promotes the proliferation and migration of CRC cells and inhibits apoptosis), the "that knockdown of HMGB1 inhibited apoptosis" should be "that knockdown of HMGB1 promoted apoptosis".

The original article has been updated.
